# Hand Recognition Obtained by Simulation of Hand Regard

**DOI:** 10.3389/fpsyg.2018.00729

**Published:** 2018-05-15

**Authors:** Takahiro Homma

**Affiliations:** Center for Industrial and Governmental Relations, University of Electro-Communications, Tokyo, Japan

**Keywords:** hand regard, cell assemblies, U-shaped developments, general movements, hand recognition, simulation

## Abstract

Eye-hand coordination of an infant is observed during the early months of their development. Hand regard, which is an example of this coordination, occurs at about 2 months. It is considered that after experiencing hand regard, an infant may recognize their own hands. However, it is unknown how an infant recognizes their hands through hand regard. Accordingly, the process by which an infant recognizes their hands and distinguishes between their hands and other objects was simulated. A simple neural network was trained with a modified real-time recurrent learning (RTRL) algorithm to deal with time-varying input and output during hand regard. The simulation results show that information about recognition of the modeled hands of an infant is stored in cell assemblies, which were self-organized. Cell assemblies appear during the phase of U-shaped developments of hand regard, and the configuration of the cell assemblies changes with each U-shaped development. Furthermore, movements like general movements (GMs) appear during the phase of U-shaped developments of hand regard.

## Introduction

Infants engage in long periods of playful self-exploration and pick up information that uniquely specifies their own body in action. This activity is considered a primary source of learning about the embodied self (Rochat, 2004). For instance, the extended hand of an infant in the posture known as the asymmetrical tonic neck reflex (ATNR) can typically be fitted into the center of the infant's visual field at about 1 month. At about 2 months, the infant can look at their own hand; in other words, “hand regard” appears. From about 3 months, sustained hand regard continues to be very common. At about 4 months, sustained hand regard is less common; instead, the infant occasionally brings the hand slowly to the object while their glance shifts from hand to object repeatedly. At about 5 months, the infant lifts the hand out of their visual field to the object quickly (i.e., “an infant's earliest reach”) (White et al., 1964). On the basis of this development of eye-hand coordination, it is considered that the infant discovers their own hands through hand regard.

Besides hand regard, another eye-hand coordination of an infant is observed during the early months of their development (von Hofsten, 2004). Infants can control the position of their arm so as to keep their hand visible (van Der Meer et al., 1995, 1997). In the first month of life, infants also show “pre-reaching” movements in which they stretch their arms toward the object but do not contact it (von Hofsten, 1984; Bhat et al., 2007). The development from pre-reaching to reaching at about 5 months described above has been explained in terms of the infant's maturing nervous system (von Hofsten, 1984). Moreover, many cases about intermodal calibration and sense of the body in infancy have been reviewed (Rochat, 2004).

From about 3 months, with sustained hand regard, an infant often clasps their hands together, over the midline (White et al., 1964). From about 5 months, the infant can grasp their right foot with their right hand and do the same with their left hand and left foot. If the infant recognizes their hands through hand regard, they may discover their feet next with recognized their own hands. Accordingly, elucidating the process for recognizing the hands is an important first step toward understanding the process for recognizing the whole body.

In the present study, a simple model for the learning of hand regard is formulated. With this model, the process by which an infant recognizes their hands and distinguishes between their hands and other objects is simulated. The present model reproduces a similar behavior as the development of visual attention for the subjects assigned to the control group of the White and Held study (White et al., 1964). Until recently, many studies on hand recognition have been reported; Some examples are infants' development of basic hand skills and visual recognition (Tomasello et al., 1993); recognition of one's own hand actions in the context of the mirror-neuron system (Rizzolatti et al., 1996); especially, theory of mind (Gallese, 2007); and attempts to model some of these processes (Oztop and Arbib, 2002). Several computational models of visual object recognition, such as VisNet (Wallis and Rolls, 1997; Tromans et al., 2011), HMAX (Riesenhuber and Poggio, 1999), and the deep neural network (Krizhevsky et al., 2012; Zeiler and Fergus, 2014), have been proposed. In these models, the output layer of a trained neural network typically contains one unit per category of the input image and implements a softmax function, which shows the probability that any of the categories are true (Kriegeskorte, 2015). In the present study, however, a learning model for recognition of one's own hand rather than an object is proposed. To recognize one's own hand, the output activities of the output units control the movements of hand; visual feedback about hand movement, corollary discharge and proprioceptive information about the hand are integrated in the present model. Several neurocomputational models adopt a brain-inspired approach to modeling the emergence of cognitive functions (i.e., language, memory, and decision making) in the brain starting from a “random” substrate. In particular, development of cell assemblies in neurobiologically realistic neural networks has been investigated (Rolls and Deco, 2002; Wennekers et al., 2006; Pulvermüller and Garagnani, 2014). Some learning models for simulating hand regard behavior were proposed. For example, in an infant model, the limitation of visual field produced hand-regard behaviors in a self-organizing manner (Yamada et al., 2010). However, the integration of visual feedback, corollary discharge and proprioceptive information were not incorporated in this model; therefore, the recognition of infant hands was not studied. In addition, a learning model that enables a robot to integrate a tactile sensation and visual feedback through hand-regard behavior was proposed (Fuke et al., 2009). The robot's hand was moved in front of the robot's face by giving a force to the hand; that is, the output activities of the output units did not control the hand movements. Therefore, corollary discharge was not incorporated in their model, and recognition of the robot hand was not studied either. The relationship between hand-regard behavior and hand recognition, which is obtained by the integration of the visual feedback, corollary discharge and proprioceptive information, has been hardly studied.

To handle hand recognition, the following points were incorporated in the proposed model. A forward model that can be utilized to determine the agent of the action has been proposed (Miall and Wolpert, 1996). To handle this function that determines the agent of the action, a simplified forward model, which produces corollary discharge, is incorporated in the present model. To deal with time-varying input and output resulting from movements of infant's hands, a real-time recurrent learning (RTRL) algorithm (Williams and Zipser, 1989; Hochreiter and Schmidhuber, 1997) is adopted. After we can recognize our own body, we feel two senses of the self: a “sense of self-ownership—the sense that it is my body that is moving; and self-agency—the sense that I am the initiator or source of the action” (Gallagher, 2000). In the process by which an infant recognizes their hands, little is known about the contribution of these two senses and the part of the brain to which they are concerned. However, since some kind of relationship is expected, the two senses proposed by Gallagher are also incorporated in the present model. This incorporation makes it possible to integrate the visual feedback, corollary discharge and proprioceptive information. In the present study, it is tested whether integrating these inputs enables hand recognition.

## Materials and methods

### Learning of hand regard

In order to create the simulation model for learning hand regard, it is necessary to know what kind of inputs an infant receives and how they process these inputs and generate the motor command to move their hands into their field of view. However, little is known of what part of the brain is related to learning of hand regard and what kind of inputs and learning rule are used to perform that learning. With respect to inputs, self-body recognition in adults can be reduced to the two senses of the self; namely, the sense of self-ownership and the sense of self-agency, which are considered to emerge mainly from the integration of visual and proprioceptive/tactile inputs and the integration of these inputs and efference copy, respectively (Jeannerod, 2003; Shimada et al., 2010). Under the supposition that an infant recognizes their own hands through learning of hand regard, it is natural to conjecture that this learning has some relation with the sense of self-ownership and the sense of self-agency. For this reason, it is hypothesized that inputs of this learning are efference copy (more precisely, corollary discharge as described in section Corollary Discharge) and visual and proprioceptive feedbacks, and a simple neural network that simulates the areas of the brain related to the sense of self-ownership and the sense of self-agency is adopted. This network is trained with a RTRL algorithm to deal with time-varying input and output resulting from movements of an infant's hands (Williams and Zipser, 1989). In the training phase, motor command errors were estimated by the difference between the position of hand and the center position of the field of view. The weights in the network were updated with these errors. By updating the weights, the network can gradually achieve hand regard. In the present study, hand regard was learnt by procedural learning to bring the infant's hands to the center of its field of view. The simulation result predicts the neuronal activity of an infant during hand regard. However, observed results of this neural activity cannot be obtained. Therefore, a time series of success rate, which is the frequency that the hand enters the center of visual field in the simulation, was compared with the observation result of the infant. The network weights were saved every 1.0 × 10^6^ time steps during the training phase. In the test phase, the success rate was calculated by these weights again. The collection of success rate calculated by the network weights saved every 1.0 × 10^6^ time steps resulted in the time series of success rate. If hand recognition is obtained, success rate increases. Therefore, a time series of success rate shows the process of hand recognition.

### Architecture

**Figure 1:**
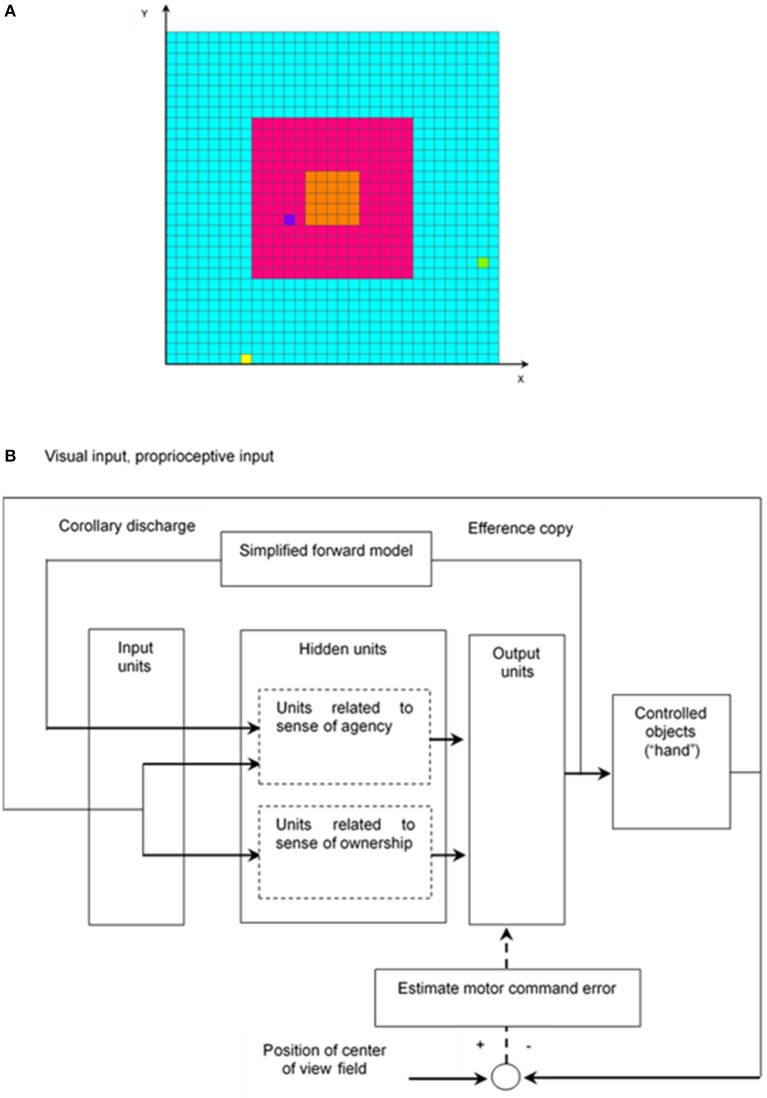
Simulation model for learning hand regard. **(A)** Infant's field of view and reachable area of infant's hands and the other object. The left hand and right hand of the infant and the other object, which are represented by the yellow, yellow-green, and blue squares, respectively, can move to the blue, red, and orange areas. The width corresponds to the length of the infant's outstretched arms. The red and orange areas are the infant's field of view, and the orange one is the center of the field of view. **(B)** Block diagram of learning hand regard.

The simulation model for learning hand regard is explained as follows. For simplicity, it is considered that the left hand and right hand of the infant and a target object are denoted by one square in a two-dimensional space (Figure 1A), and the structure of the upper limbs was omitted from the model; that is, coordinate transformations (which translate sensory inputs to motor outputs) were omitted, and a simulation calculation was executed in a two-dimensional extrinsic coordinate frame. Hereafter, in the model, one hand of the infant, both hands of the infant, an object other than the hands, and more than one object other than the hands are respectively written as “hand,” “hands,” “other,” and “others.”

The network architecture of the model, which is composed of a three-layer network, is shown in Figure 1B. The first input layer has an array of 238 input units, which receive visual input, proprioceptive input, and corollary discharge. The second hidden layer consists of 48 hidden units, which project to eight output units in the third output layer. Each hidden unit receives inputs from all input units and each output unit receives inputs from all hidden units. Four of the output units control movements of the left “hand”, and the other four control movement of the right “hand.”

The output activities of the hidden and output units are calculated by the logistic function as follows: output = 1/(1+e^−net^), where net = weighted sum of inputs. The hidden units consist of two parts. The first-part units, related to sense of agency, receive corollary discharge, visual input, and proprioceptive input from the input units and integrate them. The second-part units, related to sense of ownership, receive visual input and proprioceptive input from the input units and integrate them (section Learning of Hand Regard).

### Input

The input units receive visual input, proprioceptive input, and corollary discharge.

#### Visual input

The visual stimulus is represented on the input units. Each square in the field of view (Figure 1A) corresponds to one input unit. When the left “hand„ right “hand” or “other” moves some squares in the field of view, the input unit corresponding to the square, where the left “hand,” right “hand” or “other” stays, receives an input value of 0.5, 0.5, or 0.2, respectively in the training phase.

#### Proprioceptive input

Since hand regard is seen in blind infants (Freedman, 1964), it is assumed that the infant moves their hand into their field of view with proprioceptive information instead of visual information. Proprioceptive accuracy slightly but significantly increases with age in the age range of 8.0–24.6 years (Hearn et al., 1989); however, an infant's accuracy is unknown. It is hypothesized that the length of the infant's outstretched arms (corresponding to width in Figure 1A) is 60 cm and error in the proprioceptively perceived position of the hand is ±10 cm. Besides, a 60 × 60-cm movable area of the left “hand” and the right “hand” in Figure 1A is divided into 3 × 3 blocks (20 × 20-cm blocks). For instance, the orange, five-by-five square in the center of the field of view in Figure 1A is located at the center of the 3 × 3 blocks. Moreover, it is supposed that the infant judges the position of their hand as being at the center of a block, even if the hand is located at any other place in that block, due to error in proprioceptively perceived position; in other words, perceived positions of the left “hand” and the right “hand” take the value of any one of the central positions of the nine blocks.

#### Corollary discharge

A forward model that transforms efference copy into predicted sensory feedback (corollary discharge) has been proposed (Miall and Wolpert, 1996). To distinguish the self from the other, predicted sensory feedback and actual sensory feedback were compared (Decety and Sommerville, 2003). In particular, corollary discharge was adopted as the input instead of efference copy. Though it is possible that an infant learns the forward model during their growth, in the present study, learning of the forward model was omitted for simplicity. Corollary discharge was simply considered as the directions and distances of movement of the left “hand” and right “hand” at the next time step. The directions of that were evaluated by the calculation method described below (section Output). In contrast, since both “hands” move one square only at the next time step, the distances were ignored; therefore, corollary discharge was given by the directions of movement of the left “hand” and right “hand” at the next time step only.

The above “simplified forward model,” which was shown in Figure 1B, was applied. Efference copy in the present model is output activities of the eight output units. It was difficult to train a neural network with the output activities of the eight output units; therefore, a corollary discharge, which is given by the directions of movement of both “hands” only described above, was adopted as the input instead of efference copy.

#### Input in the test phase

The test, which consists of cases varying the visual input value of “other” and the number of “others,” was conducted. Success rate was calculated by changed visual input. In the training phase, visual input value of “hands,” visual input value of “other,” and number of “others” were 0.5, 0.2, and 1, respectively (section Visual Input). In the test phase, visual input value of “other” was 0.2 or 0.5 and number of “others” was 1, 5, or 20; that is, the test consists of 6 cases by combining 3 cases (visual input value of “other”) and 2 cases (number of “others”). Furthermore, the test was conducted without using visual input and corollary discharge. By comparing the results of success rate calculated based on the absence or presence of these inputs, it was evaluated whether hand recognition was obtained.

### Output

To reduce computational amount, movements of the left “hand” and the right “hand” were determined by the simplified population vector method (Georgopoulos et al., 1986) as follows. The preferred directions of the four output units for the left or right “hand” are upward, downward, left and right. For simplicity, it is supposed that every output unit for the left or right “hand” is not active with movements in any direction other than the preferred direction. For example, *upward-activity-left-hand* and *downward-activity-left-hand* are taken as the activities of the output units whose preferred directions for the left “hand” are upward and downward, respectively. If *upward-activity-left-hand* minus *downward-activity-left-hand* is greater than or equal to 0.8, the left “hand” moves one square upwardly, and vice versa. If the difference between those activities is less than 0.8, the left “hand” does not move. Movements of the right “hand” are determined in the same way. On the other hand, to model the control group had been reared with virtually nothing else but their own hands to view simply, the “other” moves one square randomly every 50 time steps (see section Success Rate of Hand Regard in the Training Phase).

### Relation between input and output

An efference copy is an internal copy of motor command, which consists of output activities of the output units. The efference copy was converted to corollary discharge through the simplified forward model (section Corollary Discharge). The corollary discharge then became an input of the input units at the next time step. The output activities of the output units controlled the movements of left “hand” and right “hand.” Visual and proprioceptive feedback signals of these movements also became inputs of the input units at the next time step (Figure 1B).

### Learning

The following learning algorithms have been formulated to deal with time-varying input and output. The “backpropagation through time” (BPTT) algorithm (Werbos, 1990) is an extension of the standard backpropagation algorithm for feedforward networks (Rumelhart et al., 1986). The BPTT algorithm uses the backward propagation of error information to compute the error gradient. However, because it needs to hold a whole dataset (i.e., values of input and output as well as weights at every time step), it suffers from a growing memory requirement in the case of arbitrarily long training sequences. To satisfy this need, an approximation of the BPTT algorithm, obtained by truncating the backward propagation of information to a fixed number of prior time steps (namely, a “truncated BPTT algorithm”), was proposed (Williams and Zipser, 1995). Since this approximation is, in general, only a heuristic technique, truncation errors may affect learning of hand regard.

An alternative algorithm, called “real-time recurrent learning” (RTRL) algorithm (Williams and Zipser, 1989) is a gradient-descent method that calculates the exact error gradient at every time step; namely, RTRL does not need to hold the whole dataset and does not involve truncation errors like the truncated BPTT algorithm. Therefore, RTRL algorithms was adopted and RTRL software was used (Hochreiter and Schmidhuber, 1997; Hochreiter, 2000). In advance of using RTRL software, it is necessary to prepare a set of input data and teaching signals for the training phase and input data for the test phase at every time step; however, that necessity cannot be satisfied because the positions of the left and right “hands” and “other” dynamically change. The RTRL software was therefore modified in the following way. Hand regard was learned by the procedural learning to bring both “hands” to the center of the field of view. The differences between the positions of both “hands” and the center position of the field of view were computed by using the proprioceptively perceived position (see section Proprioceptive Input). These differences were then converted to motor-command errors (i.e., differences between teaching signals and outputs) on the output units every time step on the basis of the method proposed by Kawato et al. (1987). As mentioned in section Proprioceptive Input, the movable area of the left “hand” and the right “hand” in Figure 1A was divided into 3 × 3 blocks and proprioceptively perceived positions of both “hands” take the coordinates of any one of the central positions of the nine blocks. The *x* and *y* coordinates of the central positions of the nine blocks are *x*_0_, *x*_0_+*d*, *x*_0_+2*d* and *y*_0_, *y*_0_+*d*, *y*_0_+2*d*, respectively, where *d* is the length of one side of the block. For instance, the coordinates of the central position of the central block are (*x*_0_+*d*, *y*_0_+*d*). The center of the field of view is located at the central block; therefore, its coordinates are (*x*_0_+*d*, *y*_0_+*d*). The proprioceptively perceived position of the left “hand” and right “hand” are taken as (*x*_*L*_(*t*), *y*_*L*_(*t*)) and (*x*_*R*_(*t*), *y*_*R*_(t)) at time step *t*, respectively.

The differences between the positions of both “hands” and the center position of the field of view are converted to the motor command errors at time step *t* as follows:

(1) $$\begin{array}{l} e_{0}\left(t\right)=\left(x_{0}+\text{d}-x_{L}\left(t\right)\right)/\text{d}, \end{array}$$

(2) $$\begin{array}{l} e_{1}\left(t\right)=\left(y_{0}+\text{d}-y_{L}\left(t\right)\right)/\text{d}, \end{array}$$

(3) $$\begin{array}{l} e_{2}\left(t\right)=-\left(x_{0}+\text{d}-x_{L}\left(t\right)\right)/\text{d}, \end{array}$$

(4) $$\begin{array}{l} e_{3}\left(t\right)=-\left(y_{0}+\text{d}-y_{L}\left(t\right)\right)/\text{d}, \end{array}$$

(5) $$\begin{array}{l} e_{4}\left(t\right)=\left(x_{0}+\text{d}-x_{R}\left(t\right)\right)/\text{d}, \end{array}$$

(6) $$\begin{array}{l} e_{5}\left(t\right)=\left(y_{0}+\text{d}-y_{R}\left(t\right)\right)/\text{d}, \end{array}$$

(7) $$\begin{array}{l} e_{6}\left(t\right)=-\left(x_{0}+\text{d}-x_{R}\left(t\right)\right)/\text{d}, \end{array}$$

(8) $$\begin{array}{l} e_{7}\left(t\right)=-\left(y_{0}+\text{d}-y_{R}\left(t\right)\right)/\text{d}, \end{array}$$

where *e*_0_(*t*), *e*_1_(*t*), *e*_2_(*t*), and *e*_3_(*t*) are the motor-command errors of the four output units for the left “hand” and the preferred directions of these units are right, upward, left and downward, respectively, and *e*_4_(*t*), *e*_5_(*t*), *e*_6_(*t*), and *e*_7_(*t*) are the motor command errors of the four output units for the right “hand” and the preferred directions of these units are right, upward, left and downward, respectively. These motor-command errors are zero at the central block and +1 or −1 at the other blocks. Based on these motor command errors, the overall network error at time *t* is calculated as:

(9) $$\begin{array}{l} \text{J}(\text{t})=1/2\overset{i=7}{\underset{i=0}{\sum }}{\left[e_{k}\left(t\right)\right]}^{2}. \end{array}$$

The partial derivative of the overall network error at time *t* with respect to the weight leads to the weight update. The weights in the network are updated every 10 time steps during the training phase by RTRL (Williams and Zipser, 1989).

### Comparison of observed and simulation results

**Figure 2:**
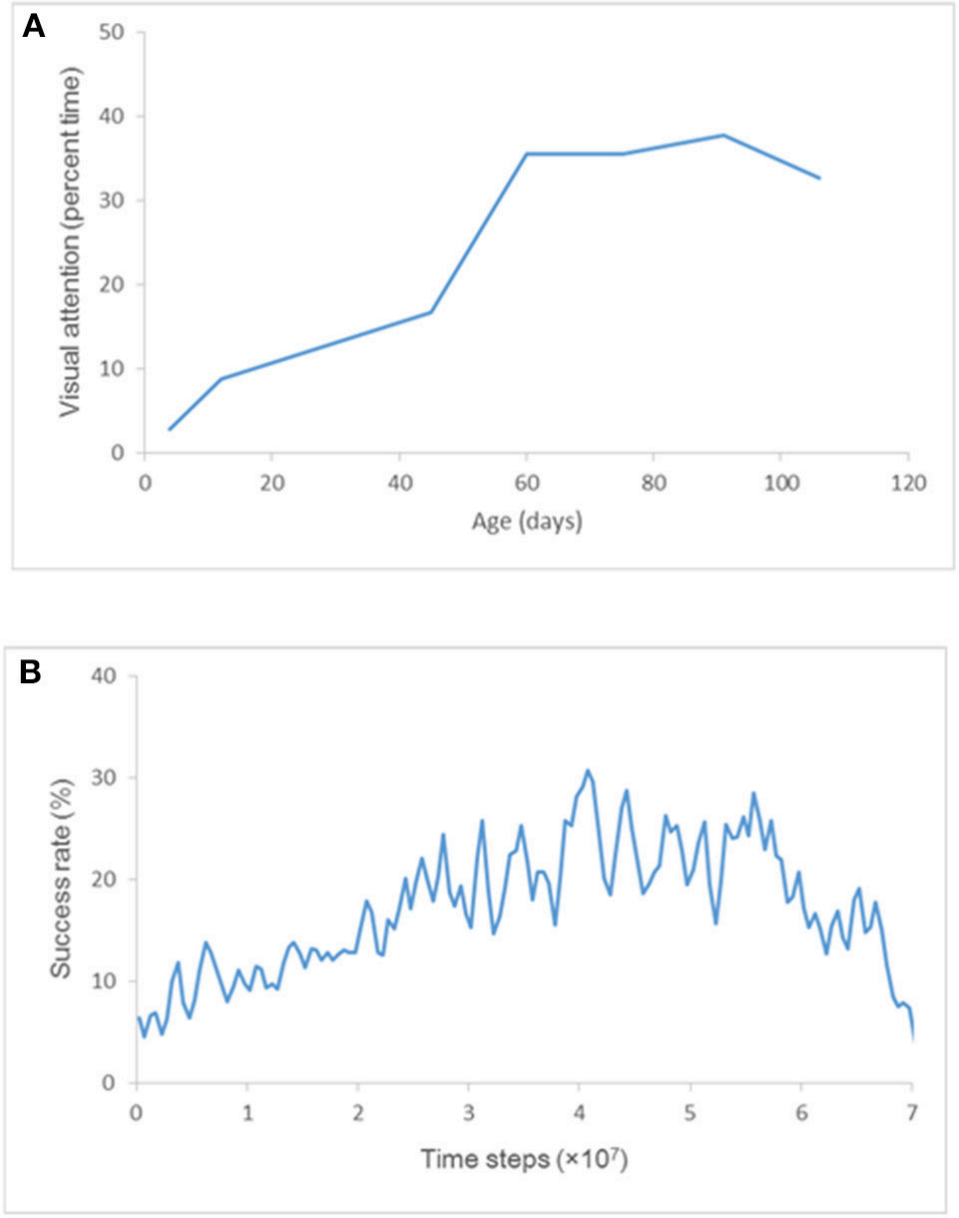
Visual attention and success rate. **(A)** Development of visual attention for the subjects assigned to the control group. Each point represents “the average of two scores taken during successive 2-week periods” (White and Held, 1966). **(B)** Plot of an ensemble average of success rates obtained by training ten times.

In a well-known study by White and Held to quantify the visual activities of an infant and grasp their spontaneous visual-motor behavior, visual attention (defined as “the state in which the infant's eyes are more than half open, their direction of gaze shifting within 30 s”) of each of several subjects was observed for 3 h every week (Figure 2A; White and Held, 1966). The subjects assigned to the control group had been reared with virtually nothing else but their own hands to view; accordingly, their visual attention could be interpreted as the frequency that they view their own hands. In fact, their visual attention increased sharply at about 2 months of age and was almost constant for the next 6 weeks or so (Figure 2A). This result can be explained by the fact that an infant begins sustained hand regard during the same period and spends considerable time watching their hands.

In the present study, the frequency that the “hands” enter the center of visual field (i.e., the frequency of receiving visual inputs of “hands” at the center of visual field) was compared with the frequency of visual attention plotted in Figure 2A (i.e., the frequency that infants hold their hands in front of their faces to view them).

After three-and-a-half months, the visible environment of these infants changed, and they could access more visual surrounds. For that reason, visual attention data after three-and-a-half months is omitted from the graph in Figure 2A.

### Success rate of hand regard

The frequency that the right or left “hand” enters the center of visual field, which is defined as success rate of hand regard, was calculated as follows.

#### Success rate of hand regard in the training phase

White and Held observed each of their subjects for 3 h (observation periods) every week (observation interval) (White and Held, 1966); therefore, the ratio of observation period to observation interval is 3/168. The observation interval in the present simulation is taken as 5 × 10^5^ time steps. Based on this ratio, the observation periods in the present simulation is approximately 1 × 10^4^ time steps.

The success rate of hand regard in the training phase was estimated as follows.

1. Count the number of time steps the right or left “hand” stayed at the orange, five-by-five square in the center of the visual field in Figure 1A for 1 × 10^4^ time steps at every 5 × 10^5^ time steps.
2. Calculate the ratio (i.e., the above number of time steps/1 × 10^4^ time steps).
3. Take the average of two ratios during 1 × 10^4^ successive time steps and define it as success rate in the training phase.

Left “hand,” right “hand” and “other” were arranged in the whole area (respectively the blue, red, and orange areas in Figure 1A) at random every 1,000 time steps. “Other” can move one square randomly every 50 time steps; that is, it can hardly move. Being arranged outside the visual field, “other” can seldom enter the field of view during 1,000 time steps. This behavior of “other” simulates the situation in which the subjects have virtually viewed nothing else but their own hands (White and Held, 1966).

#### Success rate of hand regard in the test phase

The network weights were saved every 1.0 × 10^6^ time steps during the training phase. Success rate in the test phase was estimated on the basis of the saved network weights as follows:

1. Count the number of time steps right or left “hand” stayed at the orange, five-by-five squares in the center of the visual field in Figure 1A for 1 × 10^5^ time steps using the saved network weights every 1 × 10^6^ time steps during the training phase.
2. Calculate the ratio (i.e., the above number of time steps/1 × 10^5^ time steps) and define it as success rate in the test phase.

According to the above procedure, the success rate at every 1.0 × 10^6^ time steps in the test phase was obtained. Left “hand,” right “hand” and “other” were also arranged at random every 1,000 time steps in the test phase. To average the difference between the arranged positions of left “hand,” right “hand” and “other,” the simulations were carried out for 1 × 10^5^ time steps as described above; the positions were arranged 100 times. “Others” were arranged in the whole area during the training phase. In contrast, “other” was arranged and kept in the field of view during the test phase; consequently, keeping “other” in the field of view made it more difficult to distinguish between “hand” and “other.”

## Results

### Training a neural network

A neural network was trained 10 times with weights initialized randomly in the range [−0.1, 0.1] by an RTRL algorithm, and the success rate of hand regard in the training phase was estimated. The ensemble average of the success rates obtained by the 10-times training is plotted at the midpoint of every 5 × 10^5^ time steps (i.e., 2.5 × 10^5^, 7.5 × 10^5^, 1.25 × 10^6^…) in Figure 2B. Comparing the visual attention plotted in Figure 2A and the success rate plotted in Figure 2B shows that the trained model reproduced the sharp increase in success rate just as seen in the development of visual attention at about 60 days of age.

### Cell assemblies appearing during the phase of U-shaped development

**Figure 3:**
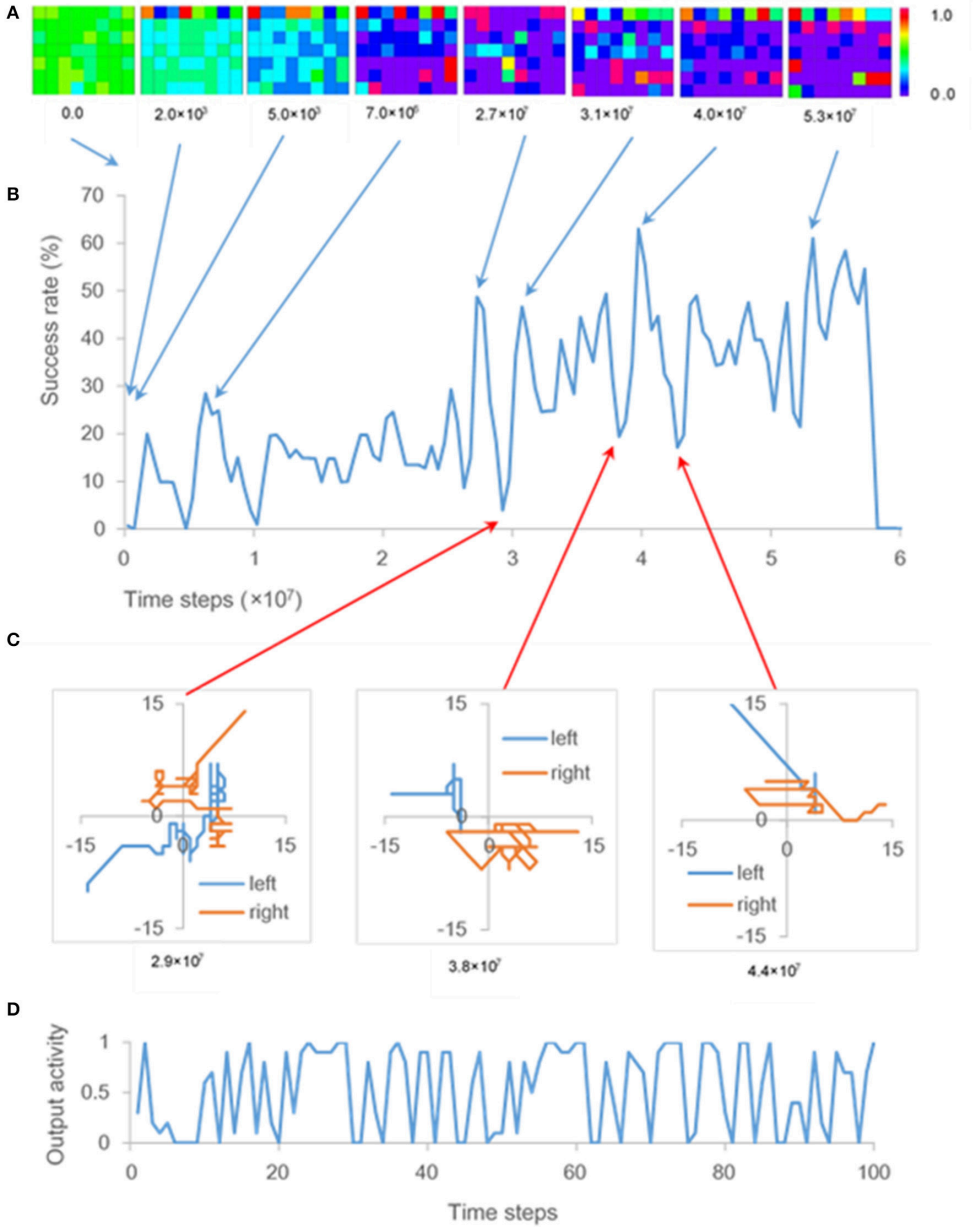
Output activity and success rate. **(A)** Output activities resulting from one of ten training times. Each panel represents output activities of the hidden and output units at 0.0, 2.0 × 10^3^, 5.0 × 10^3^, 7.0 × 10^6^, 2.7 × 10^7^, 3.1 × 10^7^, and 5.3 × 10^7^ time steps. Squares of the top line, those of lines 2–4, and those of lines 5–7 of each panel are output activities of the eight output units, 24 hidden units related to sense of ownership, and 24 hidden units related to sense of agency, respectively. **(B)** One of the time series of success rate, as described in section Cell Assemblies Appearing During the Phase of U-shaped Development, obtained by ten-times training. **(C)** Trajectories of both “hands” during a 100-time-step period at 2.9 × 10^7^ time steps, 3.8 × 10^7^ time steps, and 4.4 × 10^7^ time steps. **(D)** Time series of output activity corresponding to one of the hidden units during a 100-time-step period at 2.9 × 10^7^ time steps, when movements like GMs occurred **(C)**.

A time series of success rate (Figure 3B) indicates repeated U-shaped development. The ten-times training brings about similar patterns of U-shaped development. The ensemble average of success rates flattens the peaks of the U-shaped curve and reduces the maximum success rate to almost 30% (Figure 2B).

Since the output activities of the hidden and output units are calculated by the logistic function (section Architecture), these output activities take values from 0 to 1. The color scale in Figure 3A displays the range of these output activities. The colors of squares in each panel of Figure 3A show output activities of the output units and hidden units; that is, the red or blue square shows the output activity of output or hidden unit takes a value of 1.0 or 0.0, respectively.

Initial weights of the neural network were random in the range [−0.1, 0.1]. However, the hidden units were gradually interconnected with inhibitory weights. Most weights between the hidden units became inhibitory at 5.0 × 10^3^ time steps (Figure 3A); therefore, output activities of the hidden units were close to zero.

After the first U-shaped development, hidden units that excite each other, appeared at 7.0 × 10^6^ time steps, as shown by the red squares in Figure 3A. This result is consistent with the definition of a cell assembly (i.e., a group of neurons that are strongly coupled by excitatory synapses; Hebb, 1949). After that, the configuration of cell assemblies changed each time U-shaped developments occurred (Figure 3A). Output activities of hidden units fluctuated significantly while some inhibitory weights were transformed into excitatory ones, and the cell assembly appeared during the phase of U-shaped developments. As shown in Figure 3D, output activity of one of the hidden units fluctuated remarkably every time step. That hidden unit became one of the cell-assembly members after the fluctuation. Note that update of weights in the network during the training phase does not cause these fluctuations, because the weights were updated every 10 time steps (see section Learning). Further, since the weights in the network were not updated during the test phase, the cell assemblies that appeared in hidden units every 1.0 × 10^6^ time steps did not change during this phase.

In the present model, the fluctuations of the hidden units are projected onto the output units (Figure 1B). The fluctuations therefore affected movements of both “hands” during the phase of U-shaped developments; that is, the movements resembled general movements (GMs), which involve circular movements, moderate speed, and variable acceleration of the neck, trunk and limbs in all directions (Einspieler et al., 2007). In fact, trajectories of both “hands” during the phase of U-shaped developments indicate that movements of both “hands” were circular and zig-zag form, which are typical of GMs (Hopkins and Prechtl, 1984; Figure 3C). Note that circles became polygons because the “hands” moved through squares.

### Distinction between “hand” and “other”

**Figure 4:**
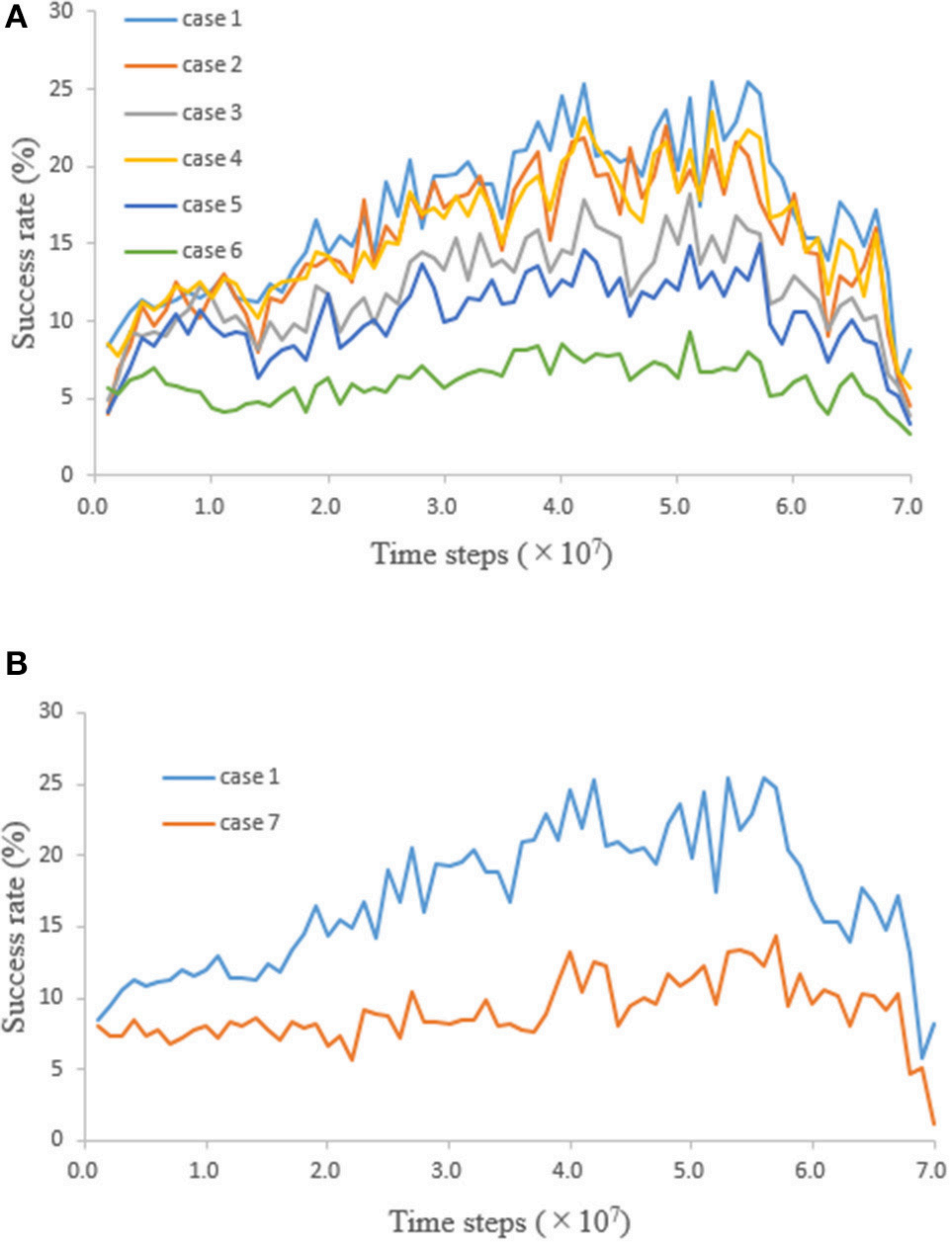
Time series of success rate obtained by testing the network. **(A)** When “other” moved some squares in the field of view, the input unit corresponding to the square where “other” stayed received a visual input value. Visual input value of “other” and number of “others” were 0.2 and 1 (case 1), 0.2 and 5 (case 2), 0.2 and 20 (case 3), 0.5 and 1 (case 4), 0.5 and 5 (case 5), and 0.5 and 20 (case 6). Visual input values of “other” in cases 1, 2, and 3 were equal to the visual input value of “other” in the training phase (i.e., 0.2). Visual input values of “other” in cases 4, 5, and 6 were equal to those of the right and left “hands” (i.e., 0.5). **(B)** Comparison of success rates in case 1 and 7.

After the network was trained, whether “hand” and “other” could be distinguished was tested. A neural network was trained 10 times with weights initialized randomly. During the training phase for each of 10 initializing weights, the network weights were saved every 1.0 × 10^6^ time steps. A time series of success rate in the test phase was obtained 10 times by testing the network with these network weights saved every 1.0 × 10^6^ time steps in response to 10 initializing weights. The test, which consists of cases varying the visual input value of “other” and the number of “others,” was conducted. The ensemble averages of the success rates obtained by the ten-times testing for each case are plotted in Figure 4A. Since case 1 is the same condition as that of the training phase, the result of case 1 was similar to the result of the training phase (Figure 2B). The network was trained by using the proprioceptively perceived positions of both “hands” (see section Learning); therefore, success rate should be constant regardless of whether the visual input exists or not. However, comparing the results for cases 1 and 4, 2 and 5, and 3 and 6 shows that success rates were reduced when the visual input value of “other” was equal to that of “hand.” Furthermore, comparing the results for cases 1, 2, and 3, or 4, 5, and 6 shows that success rates were reduced when the number of “others” increased (Figure 4A). These results are not consistent with the speculation that success rate should be constant.

From the fact that success rates changed according to the visual input condition, it is presumed that the network acquired the ability to distinguish between “hand” and “other.” When the left or right “hand” moves into the field of view on the basis of proprioceptively perceived positions, the input units receive visual input, proprioceptive input, and corollary discharge. These inputs are integrated at the hidden units. If the network acquired the ability to distinguish between them, the distinction tends to be difficult as the number of “others” increases and the input value of “other” becomes the same value as that of “hand.” This declining ability to distinguish is consistent with the test results.

In order to show that the network acquired the ability to distinguish between “hand” and “other”, the following test was further conducted (Figure 4B). The condition for case 7 is the same one for case 1 except that the visual input value of “hands,” and corollary discharge were equal to zero; that is, case 7 was the test that moved “hand” to the field of view with only proprioceptive input, without using visual input and corollary discharge. The fact that the success rate was higher in the presence of visual input and corollary discharge means that the network acquired a new ability to increase success rate by using visual input and corollary discharge. In other words, visual input, proprioceptive input, and corollary discharge were integrated and the network acquired the ability to distinguish between “hand” and “other.” By distinguishing between “hand” and “other,” it is thought that more efficient movement of “hand” became possible.

## Discussion

As explained above, the process by which an infant recognizes their hands and consequently distinguishes their hands and other objects was presented by simulating hand regard. In the present study, it was tested whether integrating the visual input, corollary discharge and proprioceptive input enables hand recognition through learning of hand regard. If trained network had acquired the ability to distinguish between “hand” and “other,” results for cases 1–5 show the success rate changed depending on the difficulty of distinguishing between “hand” and “other.” Since the network distinguished between them with the visual input, corollary discharge and proprioceptive input, it could not distinguish in case 7 without the visual input and corollary discharge. Furthermore, since the success rate of case 6 were about the same as case 7, it is estimated that the network could not distinguish in case 6 either. On the other hand, if trained network had not acquired the ability to distinguish between them, the success rates of all cases should be equal regardless of the conditions of visual input. However, there existed the difference in success rate between cases 1 and 5 and case 7. It suggests that predicted sensory feedback (corollary discharge) and actual sensory feedback (visual input) were compared in order to distinguish “hand” from “other” (section Corollary Discharge).

The difference between the results of cases 1 and 7 increased after 2.5 × 10^7^ time steps (Figure 4B). Consequently, recognition of “hands” seems to be possible after 2.5 × 10^7^ time steps. As indicated in Figure 3B, success rate in the training phase also increased after 2.5 × 10^7^ time steps, which corresponds to the onset of sustained hand regard shown in Figure 2A; therefore, it can be concluded that an infant may begin to recognize their hands during sustained hand regard.

Cell assemblies at the hidden units, where corollary discharge, visual input, and proprioceptive input were integrated, were self-organized. It has been revealed that a specific memory is stored in a cell assembly that was active during learning (Liu et al., 2012). Given that revelation, it is necessary to determine what kind of information was stored in the cell assemblies at the hidden units. Since it is thought that the network acquired the ability to distinguish between “hand” and “other” after 2.5 × 10^7^ time steps, it is concluded that the information about recognition of “hands” may have been stored in the cell assemblies after 2.5 × 10^7^ time steps. Meanwhile, the reason that cell assemblies appeared before 2.5 × 10^7^ time steps, e.g., at 7.0 × 10^6^ time steps, in Figure 3A is explained as follows. Karmiloff-Smith proposed a model incorporating a reiterative process of representational redescription with U-shaped developments of behavior (Karmiloff-Smith, 1992). Case 7 in Figure 4B was the test that moved “hand” to the field of view without recognizing “hand.” The difference in success rate between case 1 and 7 became small before 2.5 × 10^7^ time steps. Therefore, it can be inferred from this model that the information stored in the cell assemblies before 2.5 × 10^7^ time steps may have been the procedural information for bringing the “hands” to the center of the infant's field of view and may have been rewritten as information about recognition of “hands” after 2.5 × 10^7^ time steps with U-shaped developments of hand regard.

Einspieler et al. conjectured that one of the ontogenetic adaptive functions of fidgety GMs is optimal calibration of the proprioceptive system because fidgety GMs precede visual hand regard, the onset of intentional reaching, and visually controlled manipulation of objects (Einspieler et al., 2007). In contrast, the present simulation results indicate that GMs might be caused by the generation of cell assemblies with the information about recognition of hands. In the present simulation, the infant's hands were modeled simply as one point, which was moved by output activities of four output units; therefore, a simple comparison between simulated movements and GMs may be not appropriate. However, if the fluctuations of output activity resulting from cell assemblies occur in a certain part of an infant's brain and project onto the area controlling their hands and arms as the present simulation, complicated movements like GMs may appear during the process of hand regard. Fidgety GMs disappear around 20 weeks post-term (Prechtl, 2001), and hand regard disappears around the same time (White et al., 1964). And that concurrence is consistent with the results of the present simulation.

The overall network error (Equation 9) is minimized in RTRL algorithm; consequently, one of the local minimum of this error corresponds to the emergence of cell assemblies in the network. What these assemblies change after each U-shaped development corresponds to the transition to another local minimum. However, the mechanisms that lead to the emergence of cell assemblies are still incompletely understood; in particular, little is known about why the hidden units were gradually interconnected with inhibitory weights (Figure 3A). Furthermore, after the small-scale U-shaped developments other than the wide U-shaped developments explained in section Cell Assemblies Appearing during the Phase of U-shaped Development, a part of the configuration of the cell assembly has sometimes changed. The effect of size of U-shaped development on this change has not been elucidated. Since observation period of visual attention is long (every 1 week) (section Comparison of Observed and Simulation Results), it was impossible to confirm whether U-shaped development occurred. Besides, observing the neuronal activity of an infant during hand regard has not been obtained. Therefore, it has not been achieved to compare simulation predictions and experimental results in detail. It is required to investigate the information stored in the cell assemblies and the relation between cell assemblies and U-shaped developments.

The hidden units were divided into two parts simulating the two brain regions implicated in the sense of self-ownership and the sense of self-agency. Frequency of appearance of cell assemblies in both parts depended on the values of the initialized weights. The contribution of both parts to distinction between “hand” and “other” has still not been elucidated. Additionally, it is necessary to verify whether simulating hand regard by using a learning algorithm other than RTRL would show the generation of cell assemblies.

Structures of upper limbs, movements of the neck and eyeball, and the asymmetrical tonic neck reflex (ATNR) were omitted from the proposed model. Improving the model to handle tactile input may elucidate the process of self-body recognition with recognized hands through hand regard. In addition, adding binocular depth cues and movements of the neck and eyeball to the model may make it possible to simulate an infant's earliest reach with alternating glances.

## Author contributions

The author confirms being the sole contributor of this work and approved it for publication.

### Conflict of interest statement

The author declares that the research was conducted in the absence of any commercial or financial relationships that could be construed as a potential conflict of interest. The reviewer, DB, and handling Editor declared their shared affiliation.
